# Quantum Cournot Triopoly Game with Heterogeneous Expectations: Dynamics and Chaos Control with Isoelastic Demand

**DOI:** 10.3390/e28070788

**Published:** 2026-07-12

**Authors:** Longfei Wei, Shouli Wang, Jing Wang

**Affiliations:** School of Management, Liaoning Normal University, Dalian 116081, China; wlf@lnnu.edu.cn (L.W.); wsl_lnnu@163.com (S.W.)

**Keywords:** quantum cournot triopoly, heterogeneous expectations, bifurcation and chaos, isoelastic demand, chaos control

## Abstract

This paper investigates how quantum entanglement and heterogeneous expectations jointly affect the stability, complexity, and controllability of a Cournot triopoly with isoelastic demand. Based on the Li–Du–Massar quantization scheme, we construct a discrete-time quantum Cournot triopoly in which three firms adopt different updating mechanisms: boundedly rational adjustment, naïve and adaptive expectations. The quantum boundary equilibrium and the unique interior quantum Nash equilibrium are derived explicitly. By linearizing the resulting three-dimensional nonlinear map and applying the Jury criterion, we obtain analytical local stability conditions for the interior equilibrium. The results show that increasing the entanglement level reduces the admissible range of the adjustment speed, thereby shrinking the stability domain and making the market dynamics more prone to bifurcation and chaos. Numerical simulations further reveal a typical transition from stable convergence to flip bifurcation, period-doubling cascades, chaotic attractors, and sensitive dependence on initial conditions. Finally, a control parameter is introduced to rescale the effective adjustment speed of the boundedly rational firm. This mechanism preserves the equilibrium set while restoring convergence to a stable fixed point once the control intensity exceeds a critical threshold. The findings highlight the joint role of entanglement, expectation heterogeneity, and nonlinear demand in shaping complex quantum oligopoly dynamics.

## 1. Introduction

Rapid progress in quantum information science and quantum technologies has stimulated the development of quantum game theory as a new analytical framework for strategic decision making, in which superposition, entanglement, and non-classical correlations can be incorporated into players’ information and action spaces. Since the seminal Eisert–Wilkens–Lewenstein quantization scheme, quantum strategies have been shown to reshape equilibrium outcomes and strategic incentives relative to their classical counterparts [1,2,3]. Subsequent studies have further clarified the methodological foundations and interpretation of quantum games and have shown their value for modeling complex decision problems with interacting agents [4,5]. At the same time, robustness issues such as time dependence and quantum noise have also been examined, supporting the use of quantum games in more realistic environments [6,7]. Beyond methodological developments, quantum-game models have increasingly been applied in economics and management, including channel decisions and cooperation problems, indicating their potential as a useful modeling language for market competition [8].

Cournot quantity competition is one of the cornerstones of oligopoly theory [9]. In dynamic Cournot models, firms adjust quantities iteratively rather than jumping directly to equilibrium, and this process may generate rich nonlinear phenomena such as instability, bifurcation, and chaos [10,11]. The classical literature on nonlinear oligopolies has provided systematic tools for analyzing these issues, including local stability conditions based on the Jury criterion [12,13]. In the quantum domain, Li et al. proposed a continuous-variable quantization scheme that preserves continuous strategic variables and showed that entanglement can effectively induce more cooperative outcomes even under selfish behavior [14]. Later work further extended quantum Cournot competition to richer settings, including bounded rationality, heterogeneous expectations, quadratic costs, and alternative learning mechanisms [15,16,17,18,19,20,21,22]. Demand curvature has also been incorporated into quantum Cournot dynamics; in particular, isoelastic demand introduces stronger nonlinear feedback and materially changes the resulting stability and global dynamics [23,24,25,26].

In some oligopolistic markets, competition may be better represented by three major firms rather than by two. Compared with duopoly, triopoly introduces an additional strategic feedback channel and increases system dimensionality, which is well known to generate richer instability mechanisms and more intricate transitions among steady, periodic, and chaotic regimes [27,28,29,30]. Classical studies on triopoly dynamics have documented explicit stability regions, multistability, and complex adjustment processes under alternative behavioral assumptions [31,32,33]. In the quantum setting, however, triopoly models remain relatively limited, especially when heterogeneous expectation rules and nonlinear demand are considered simultaneously [34].

Another important modeling feature is the heterogeneity of expectations and updating rules. In actual markets, firms differ in information-processing ability, managerial sophistication, and learning behavior [35,36]. Heterogeneous adjustment is therefore more realistic than the assumption of homogeneous perfect rationality. In both classical and quantum Cournot dynamics, bounded rationality, adaptive expectations, memory, and other learning mechanisms have been shown to reshape stability boundaries and routes to complexity [19,37,38]. In addition, the form of market demand plays a crucial role in determining the nonlinearity embedded in firms’ payoffs. Compared with linear demand, isoelastic demand produces non-constant marginal effects and can significantly alter equilibrium properties, local stability, and bifurcation thresholds [39,40,41,42]. These observations motivate the study of a quantum Cournot triopoly with heterogeneous expectations and isoelastic demand.

When chaotic fluctuations arise, control and stabilization become practically important because chaotic output paths are irregular, highly sensitive to initial conditions, and undesirable for planning, regulation, and risk management [10]. A substantial literature has therefore developed chaos-control approaches in Cournot-type models [43,44,45], including feedback control, delayed feedback control [46], and related stabilization schemes in classical and quantum settings [47,48,49]. These studies suggest that effective control mechanisms can suppress chaotic dynamics without necessarily altering the equilibrium structure of the model.

Despite these developments, several issues remain insufficiently explored. First, most existing quantum Cournot models focus on duopoly competition, whereas the quantum dynamics of triopoly markets with an additional strategic feedback channel have received less attention. Although quantum Cournot triopoly dynamics with heterogeneous players have been considered in recent studies [34], the joint role of isoelastic demand, heterogeneous expectation rules, and equilibrium-preserving chaos control has not yet been systematically examined. Second, although heterogeneous expectations and nonlinear demand have each been studied in Cournot-type models, their combined effects in a continuous-variable quantum triopoly framework remain unclear. Third, existing chaos-control studies mainly emphasize numerical stabilization, while the relationship between adjustment-speed control, equilibrium preservation, and local stability properties still deserves further clarification. This study is relevant to the scope of Entropy because it shows how quantum entanglement and heterogeneous information-processing rules jointly reshape the stability, complexity, and controllability of a nonlinear economic game. In this sense, the model provides an information- and complexity-oriented perspective on quantum oligopoly dynamics.

A closely related dynamic quantum Cournot triopoly with heterogeneous players was studied in our previous work [34], where boundedly rational, naïve, and adaptive players were incorporated into a continuous-variable quantum triopoly framework. However, the present paper differs from that study in both model structure and analytical focus. The key difference lies in the introduction of isoelastic demand. Under the inverse demand function $p=\frac{1}{Q}$, the market price generates a state-dependent nonlinear feedback, and the best-response rules of the naïve and adaptive firms become square-root mappings. This feature changes the feasible strategy domain, the equilibrium conditions, the marginal-profit structure, the Jacobian matrix, and the Jury stability inequalities of the dynamic system. Therefore, the results of the previous heterogeneous quantum triopoly model cannot be directly applied to the present setting. The original scientific contribution of this paper is to clarify how isoelastic demand reshapes the feasible strategy domain, equilibrium and stability conditions, bifurcation mechanism, and chaos-control threshold of a heterogeneous quantum Cournot triopoly.

Motivated by the above considerations, this paper develops a discrete-time dynamical model of a continuous-variable quantum Cournot triopoly with heterogeneous expectations and isoelastic demand. Firm 1 adopts a boundedly rational adjustment rule, firm 2 follows a naïve best-response rule, and firm 3 updates according to adaptive expectations. Within this framework, we derive the quantum boundary equilibrium and the unique interior quantum Nash equilibrium, analyze local stability by means of the Jacobian matrix and the Jury criterion, and investigate the effects of entanglement and adjustment speed on bifurcation and chaos. We further introduce a simple control parameter to suppress chaotic fluctuations while keeping the equilibrium set unchanged.

Compared with the existing literature, the main contributions of this paper are threefold. First, this paper derives a new nonlinear discrete-time system for a heterogeneous continuous-variable quantum Cournot triopoly under isoelastic demand. Unlike the previous heterogeneous quantum triopoly benchmark, isoelastic demand generates square-root best-response mappings and imposes additional feasibility requirements on the positive strategy domain. Second, we derive the quantum boundary equilibrium and the interior quantum Nash equilibrium under this new nonlinear structure, verify the relevant optimality conditions, and obtain explicit local stability conditions by applying the Jury criterion to the corresponding Jacobian matrix. These results clarify how nonlinear demand feedback, quantum entanglement, and heterogeneous expectations jointly determine the stability threshold of the interior equilibrium. Third, we analyze the bifurcation route, chaotic dynamics, sensitivity to initial conditions, and adjustment-speed control mechanism in the isoelastic-demand system. The control scheme preserves the equilibrium set while restoring stability by reducing the effective adjustment speed of the boundedly rational firm.

The remainder of this paper is organized as follows. Section 2 introduces the classical Cournot triopoly benchmark, formulates its continuous-variable quantum extension, and constructs the discrete-time heterogeneous dynamical system. Section 3 derives the equilibrium points and establishes local stability conditions of the quantum Nash equilibrium. Section 4 presents numerical simulations, including stability regions, bifurcation diagrams, maximum Lyapunov exponents, phase portraits, and sensitivity to initial conditions. Section 5 proposes a control scheme and verifies its effectiveness numerically. Section 6 concludes the paper.

## 2. The Model

### 2.1. The Classical Cournot Triopoly Game

We consider a triopoly oligopoly market with three firms, j=1,2,3. In each period, firms simultaneously choose quantities $q_{j}$ of a homogeneous product. The demand is assumed to be isoelastic, so the market price is determined by total quantity as [39,40]

(1) $$p=f\left(q\right)=\frac{1}{q_{1}+q_{2}+q_{3}}.$$

Each firm is assumed to face a linear cost function with a common constant marginal cost c>0

(2) $$C_{j}\left(q_{j}\right)=cq_{j},\;j=1,2,3.$$

Accordingly, firm *j*’s profit can be written as

(3) $$\pi_{j}\left(q_{1},q_{2},q_{3}\right)=q_{j}\left(p-c\right)=q_{j}\left(\frac{1}{q_{1}+q_{2}+q_{3}}-c\right),\;j=1,2,3.$$

This classical setting provides the benchmark for the quantum Cournot triopoly developed below.

### 2.2. Quantum Form of the Cournot Triopoly Game

Following the continuous-variable quantization proposed by Li et al. [14] and further developed for Cournot-type competition [15], we construct a quantum extension of the classical Cournot triopoly. The game is initialized in the three-mode vacuum state ${|\mathrm{vac}\rangle }_{1}⊗{|\mathrm{vac}\rangle }_{2}⊗{|\mathrm{vac}\rangle }_{3}$. A publicly known entangling operator $\hat{J}\left(\gamma \right)$ is first applied to generate correlations among the three strategic modes. Each firm *j* then implements its local unitary strategy ${\hat{D}}_{j}\left(x_{j}\right)$, where $x_{j}\geq 0$ is the firm’s continuous quantum strategy parameter, and $x_{j}>0$ corresponds to an interior strategy. Finally, the disentangling operation ${\hat{J}}^{†}\left(\gamma \right)$ is performed before measurement. Under this standard entangle–operate–disentangle protocol, the final pre-measurement state reads

(4) $$|\psi_{f}\rangle ={\hat{J}}^{†}\left(\gamma \right)\left({\hat{D}}_{1}\left(x_{1}\right)⊗{\hat{D}}_{2}\left(x_{2}\right)⊗{\hat{D}}_{3}\left(x_{3}\right)\right)\hat{J}\left(\gamma \right){|\mathrm{vac}\rangle }_{1}⊗{|\mathrm{vac}\rangle }_{2}⊗{|\mathrm{vac}\rangle }_{3}.$$

The parameter γ≥0 is the squeezing parameter and serves as a natural measure of the entanglement strength in this scheme.

In the economic interpretation of this model, γ can be regarded as an abstract measure of strategic correlation among firms rather than as explicit collusion. A larger γ implies that firms’ effective strategic variables are more strongly interconnected through the quantum interaction structure. Such correlation may represent common market signals, shared demand information, technological spillovers, or other forms of inter-firm linkage in an oligopolistic market. This interpretation is consistent with the continuous-variable quantum-game framework [14] and with the quantum Cournot literature in which entanglement mixes the strategic impacts across firms [16,17,18,34]. When γ=0, the model reduces to the classical independent-strategy case; as γ increases, the cross-strategy influence embodied in the effective quantum quantities becomes stronger.

To extract economically interpretable decisions, we measure the quadratures of the output modes. Let ${\hat{X}}_{j}=\left({\hat{a}}_{j}^{†}+{\hat{a}}_{j}\right)/\sqrt{2}$ denote the position operator of firm *j*, where ${\hat{a}}_{j}^{†}$ and ${\hat{a}}_{j}$ are the creation and annihilation operators of the *j*th mode. The multi-mode entangling operator is given by

(5) $$\hat{J}\left(\gamma \right)=\exp\left\{-\gamma \sum_{\begin{array}{c} i,j=1\\ i\neq j \end{array}}^{3}\left({\hat{a}}_{i}^{†}{\hat{a}}_{j}^{†}-{\hat{a}}_{i}{\hat{a}}_{j}\right)\right\}.$$

Under the action of $\hat{J}\left(\gamma \right)$, a local displacement ${\hat{D}}_{j}\left(x_{j}\right)$ of firm *j* is transformed into a coupled operation that affects all three modes, namely,

(6) $${\hat{J}}^{†}\left(\gamma \right){\hat{D}}_{j}\left(x_{j}\right)\hat{J}\left(\gamma \right)=\exp\left\{-ix_{j}\left[\frac{1}{3}\left(e^{2\gamma }+2e^{-\gamma }\right){\hat{P}}_{j}+\sum_{\begin{array}{c} i=1\\ i\neq j \end{array}}^{3}\frac{1}{3}\left(e^{2\gamma }-e^{-\gamma }\right){\hat{P}}_{i}\right]\right\},$$

where ${\hat{P}}_{j}=i\left({\hat{a}}_{j}^{†}-{\hat{a}}_{j}\right)/\sqrt{2}$ is the corresponding momentum operator. This form highlights that entanglement mixes the strategic impacts across firms, so that each firm’s effective action depends not only on its own strategy but also on its rivals’ strategies [16,17,18,34].

Since the transformed displacement operators act on the joint three-mode Hilbert space, the final state is more precisely written as a single multi-mode exponential operator acting on the product vacuum state

(7) $$\begin{array}{cc} |\psi_{f}\rangle = & \;\exp\left\{-i\sum_{j=1}^{3}x_{j}\left[\frac{1}{3}\left(e^{2\gamma }+2e^{-\gamma }\right){\hat{P}}_{j}+\frac{1}{3}\left(e^{2\gamma }-e^{-\gamma }\right)\sum_{\begin{array}{c} i=1\\ i\neq j \end{array}}^{3}{\hat{P}}_{i}\right]\right\}\times {|\mathrm{vac}\rangle }_{1}⊗{|\mathrm{vac}\rangle }_{2}⊗{|\mathrm{vac}\rangle }_{3}. \end{array}$$

Based on the output transformation induced by the quantum measurement, the effective quantum quantities can be written as

(8) $$q_{j}=x_{j}\frac{1}{3}\left(e^{2\gamma }+2e^{-\gamma }\right)+\sum_{\begin{array}{c} i=1\\ i\neq j \end{array}}^{3}x_{i}\frac{1}{3}\left(e^{2\gamma }-e^{-\gamma }\right),\;j=1,2,3,$$

and the aggregate quantity is

(9) $$q=\sum_{j=1}^{3}x_{j}e^{2\gamma }.$$

Substituting these effective quantum quantities into the classical profit function, the quantum profit of firm *j* is

(10) $$\begin{array}{cc} \pi_{j}^{Q}\left(x_{1},x_{2},x_{3}\right)\; & =\pi_{j}\left(q_{1},q_{2},q_{3}\right)\\ \; & =\frac{1}{3}\left[x_{j}\left(e^{2\gamma }+2e^{-\gamma }\right)+\sum_{\begin{array}{c} i=1\\ i\neq j \end{array}}^{3}x_{i}\left(e^{2\gamma }-e^{-\gamma }\right)\right]\left(\frac{e^{-2\gamma }}{x_{1}+x_{2}+x_{3}}-c\right). \end{array}$$

To verify that the first-order conditions characterize a maximum rather than merely a stationary point, we further examine the second-order condition. From Equation (10), the marginal profit of firm *j* with respect to its own strategy is

$$\frac{\partial \pi_{j}^{Q}}{\partial x_{j}}=\frac{e^{-3\gamma }\sum_{i\neq j}^{3}x_{i}}{{\left(x_{1}+x_{2}+x_{3}\right)}^{2}}-\frac{c(e^{2\gamma }+2e^{-\gamma })}{3}.$$

Taking the derivative again with respect to $x_{j}$, we obtain

$$\frac{\partial^{2}\pi_{j}^{Q}}{\partial x_{j}^{2}}=-\frac{2e^{-3\gamma }\sum_{\begin{array}{c} i\neq j \end{array}}^{3}x_{i}}{{\left(x_{1}+x_{2}+x_{3}\right)}^{3}}<0,$$

whenever $\sum_{i\neq j}^{3}x_{i}>0$. Therefore, given positive rivals’ strategies, $\pi_{j}^{Q}$ is strictly concave in $x_{j}$, and the first-order condition is sufficient for firm *j*’s interior profit maximization. Solving the simultaneous first-order conditions $\partial \pi_{j}^{Q}/\partial x_{j}=0$ for j=1,2,3, we obtain the unique interior quantum Nash equilibrium of the static quantum game

(11) $$x_{1}^{*}=x_{2}^{*}=x_{3}^{*}=\frac{2e^{-3\gamma }}{3c\left(e^{2\gamma }+2e^{-\gamma }\right)}.$$

At this equilibrium, the corresponding equilibrium outputs are

(12) $$q_{1}^{*}=q_{2}^{*}=q_{3}^{*}=\frac{2e^{-\gamma }}{3c\left(e^{2\gamma }+2e^{-\gamma }\right)}.$$

The corresponding profits are

(13) $$\pi_{1}^{Q}\left(x_{1}^{*},x_{2}^{*},x_{3}^{*}\right)=\pi_{2}^{Q}\left(x_{1}^{*},x_{2}^{*},x_{3}^{*}\right)=\pi_{3}^{Q}\left(x_{1}^{*},x_{2}^{*},x_{3}^{*}\right)=\frac{1}{3(1+2e^{-3\gamma })}.$$

The static quantum Nash equilibrium is obtained from the first-order conditions, with the above second-order condition confirming that the equilibrium strategy profile corresponds to interior profit maximization. Substituting this strategy profile into the effective-output equation yields the corresponding symmetric equilibrium outputs, and substituting it into the quantum profit function gives the equilibrium profits. In particular, when γ=0, entanglement disappears and the quantum formulation reduces to the classical Cournot triopoly benchmark. As γ increases, entanglement strengthens the strategic correlation among firms and can improve equilibrium payoffs.

### 2.3. The Dynamic Quantum Cournot Triopoly Game with Heterogeneous Players

To characterize the intertemporal evolution of the quantum Cournot triopoly under bounded rationality and heterogeneous expectations, we consider a discrete-time adjustment process. Let $x_{j}\left(t\right)$ (j=1,2,3) denote firm *j*’s quantum strategic variable at period *t*. The three firms are assumed to adopt heterogeneous decision and learning mechanisms, reflecting different levels of information processing and behavioral sophistication. The specific combination of bounded rationality, naïve expectation, and adaptive expectation is chosen as a benchmark heterogeneous structure for a triopoly market. These three rules represent three common behavioral types: local gradient adjustment based on marginal profit, instantaneous best response under static beliefs about rivals, and partial adjustment with learning inertia. Such heterogeneity is consistent with the view that firms differ in information-processing ability and learning behavior [35,36], and with the Cournot-dynamics literature showing that heterogeneous expectations can reshape stability and complexity [19,37]. The present assignment allows the three-firm model to incorporate these representative behavioral rules simultaneously. For notational convenience, we introduce the positive constant $\alpha =e^{2\gamma }+2e^{-\gamma }>0$, which repeatedly appears in the subsequent expressions.

Firm 1 is assumed to be boundedly rational and to update its strategy according to a gradient-based rule using only local marginal-profit information. Specifically, its adjustment dynamics are described by Equation (14),

(14) $$x_{1}\left(t+1\right)=x_{1}\left(t\right)+vx_{1}\left(t\right)\frac{\partial \pi_{1}^{Q}}{\partial x_{1}},$$

where v>0 is the speed of adjustment. This rule implies that firm 1 increases (decreases) its next-period strategy whenever the marginal profit $\partial \pi_{1}^{Q}/\partial x_{1}$ is positive (negative).

Firm 2 is assumed to form naïve expectations. When making its decision at time *t*, it expects its competitors to keep their current strategies unchanged in period t+1. Accordingly, under the nonnegative strategy constraint, firm 2 chooses $x_{2}\left(t+1\right)$ as the best response to the current strategies of firms 1 and 3, as stated in Equation (15).

(15) $$x_{2}\left(t+1\right)=\arg\max_{x_{2}\geq 0}\pi_{2}^{Q}\left(x_{1}\left(t\right),x_{2},x_{3}\left(t\right)\right).$$

The first-order condition gives the following interior best-response branch:

(16) $$x_{2}\left(t+1\right)=\sqrt{\frac{3e^{-3\gamma }\;\left(x_{1}\left(t\right)+x_{3}\left(t\right)\right)}{c\alpha }}-x_{1}\left(t\right)-x_{3}\left(t\right).$$

Firm 3 is assumed to follow an *adaptive-expectations* mechanism. Rather than jumping directly to its instantaneous best response, it partially adjusts its strategy toward that best-response level. This behavior is modeled by Equation (17),

(17) $$x_{3}\left(t+1\right)=\left(1-w\right)x_{3}\left(t\right)+w\left(\sqrt{\frac{3e^{-3\gamma }\;\left(x_{1}\left(t\right)+x_{2}\left(t\right)\right)}{c\alpha }}-x_{1}\left(t\right)-x_{2}\left(t\right)\right).$$

where 0<w<1 denotes the adaptive adjustment coefficient: a smaller *w* reflects stronger inertia, whereas a larger *w* implies faster adjustment toward the target level.

It should be noted that Equations (16) and (17) describe the interior best-response branch of the corresponding one-period optimization problem. For firm 2, the interior response is positive if and only if $0<x_{1}\left(t\right)+x_{3}\left(t\right)<\frac{3e^{-3\gamma }}{c\alpha }.$

If this condition is violated, the interior branch is no longer feasible. In that case, the constrained nonnegative best response is located at the boundary of the strategy space, namely $x_{2}\left(t+1\right)=0$. Economically, this corresponds to a situation in which the rivals’ strategic levels are sufficiently high that firm 2 optimally chooses zero output in that period. Similarly, the best-response target of firm 3 is positive when $0<x_{1}\left(t\right)+x_{2}\left(t\right)<\frac{3e^{-3\gamma }}{c\alpha }.$

Outside this interior feasible region, the constrained response would also be projected onto the nonnegative boundary. Since the present paper focuses on the interior equilibrium and its local stability, the subsequent analysis is conducted on the positive interior branch of the dynamic system. At the interior equilibrium $E_{2}$, one has

$$x_{1}^{*}+x_{3}^{*}=x_{1}^{*}+x_{2}^{*}=\frac{4e^{-3\gamma }}{3c\alpha }<\frac{3e^{-3\gamma }}{c\alpha },$$

so the interior best-response conditions are satisfied. Therefore, there exists a sufficiently small feasible neighborhood of $E_{2}$ in which the square-root best-response mappings remain positive and the local Jacobian and Jury stability analysis are well defined.

Combining the updating rules of the three firms, the heterogeneous quantum Cournot triopoly can be written as follows:

(18) $$\left\{\begin{array}{c} x_{1}\left(t+1\right)=x_{1}\left(t\right)+v\;x_{1}\left(t\right)\;\left(\frac{e^{-3\gamma }\;\left(x_{2}\left(t\right)+x_{3}\left(t\right)\right)}{{\left(x_{1}\left(t\right)+x_{2}\left(t\right)+x_{3}\left(t\right)\right)}^{2}}-\frac{c\alpha }{3}\right),\\ x_{2}\left(t+1\right)=\sqrt{\frac{3e^{-3\gamma }\;\left(x_{1}\left(t\right)+x_{3}\left(t\right)\right)}{c\alpha }}-x_{1}\left(t\right)-x_{3}\left(t\right),\\ x_{3}\left(t+1\right)=\left(1-w\right)x_{3}\left(t\right)+w\;\left(\sqrt{\frac{3e^{-3\gamma }\;\left(x_{1}\left(t\right)+x_{2}\left(t\right)\right)}{c\alpha }}-x_{1}\left(t\right)-x_{2}\left(t\right)\right) \end{array}\right.$$

## 3. Quantum Equilibrium Points and Local Stability

Given the discrete-time dynamical system (18), an equilibrium (fixed point) is a state $E=(x_{1}^{*},x_{2}^{*},x_{3}^{*})$ such that $x_{i}\left(t+1\right)=x_{i}\left(t\right)=x_{i}^{*}$ for i=1,2,3. Imposing this fixed-point condition on the three updating equations in (18) yields two equilibria, denoted by $E_{1}$ and $E_{2}$, as given in Equation (19)

(19) $$\begin{array}{cc} E_{1} & =(0,\frac{3e^{-3\gamma }}{4c\alpha },\frac{3e^{-3\gamma }}{4c\alpha }),\\ E_{2} & =\left(x_{1}^{*},x_{2}^{*},x_{3}^{*}\right)=\left(\frac{2e^{-3\gamma }}{3c\alpha },\frac{2e^{-3\gamma }}{3c\alpha },\frac{2e^{-3\gamma }}{3c\alpha }\right). \end{array}$$

It follows that $E_{1}$ is a quantum boundary equilibrium point, while $E_{2}$ is the unique interior quantum Nash equilibrium point. To study the local stability of the quantum equilibrium points in the three-dimensional system (18), we compute the Jacobian matrix of the map as follows:

(20) $$J\left(x_{1},x_{2},x_{3}\right)=\left(\begin{array}{ccc} 1-\frac{vc\alpha }{3}+ve^{-3\gamma }\frac{\left(x_{2}+x_{3}\right)\left(x_{2}+x_{3}-x_{1}\right)}{{\left(x_{1}+x_{2}+x_{3}\right)}^{3}}\; & ve^{-3\gamma }\frac{x_{1}\left(x_{1}-x_{2}-x_{3}\right)}{{\left(x_{1}+x_{2}+x_{3}\right)}^{3}}\; & ve^{-3\gamma }\frac{x_{1}\left(x_{1}-x_{2}-x_{3}\right)}{{\left(x_{1}+x_{2}+x_{3}\right)}^{3}}\\ \frac{1}{2}\sqrt{\frac{3e^{-3\gamma }}{c\alpha (x_{1}+x_{3})}}-1\; & 0\; & \frac{1}{2}\sqrt{\frac{3e^{-3\gamma }}{c\alpha (x_{1}+x_{3})}}-1\\ w\left[\frac{1}{2}\sqrt{\frac{3e^{-3\gamma }}{c\alpha (x_{1}+x_{2})}}-1\right]\; & w\left[\frac{1}{2}\sqrt{\frac{3e^{-3\gamma }}{c\alpha (x_{1}+x_{2})}}-1\right]\; & 1-w \end{array}\right).$$

**Proposition** **1.** *The quantum boundary equilibrium $E_{1}$ is unstable*.

**Proof.**  Substituting $E_{1}$ into the Jacobian matrix $J(x_{1},x_{2},x_{3})$ in Equation (20), we obtain the linearization at $E_{1}$ as the diagonal matrix in (21)

(21) $$J\left(E_{1}\right)=\left(\begin{array}{ccc} 1+\frac{vc\alpha }{3}\; & 0\; & 0\\ 0\; & 0\; & 0\\ 0\; & 0\; & 1-w \end{array}\right).$$

Therefore, the eigenvalues are directly given by the diagonal entries,

$$\begin{array}{c} \lambda_{1}=1+\frac{vc\alpha }{3},\;\lambda_{2}=0,\;\lambda_{3}=1-w. \end{array}$$

Since v>0, c>0 and $\alpha =e^{2\gamma }+2e^{-\gamma }>0$, it follows that

$$\lambda_{1}=1+\frac{vc\alpha }{3}>1.$$

For a discrete-time dynamical system, the presence of any eigenvalue satisfying |λ|>1 implies that the fixed point is unstable. Hence, the quantum boundary equilibrium $E_{1}$ is unstable. □

Remark. The above instability result depends on the symmetric marginal-cost assumption. If the cost function is extended to $C_{j}\left(q_{j}\right)=c_{j}q_{j}$, $c_{j}>0$, a boundary equilibrium with firm 1 inactive and firms 2 and 3 active can be written as

$$E_{1}^{a}=\left(0,\frac{3e^{-3\gamma }c_{3}}{\alpha {\left(c_{2}+c_{3}\right)}^{2}},\frac{3e^{-3\gamma }c_{2}}{\alpha {\left(c_{2}+c_{3}\right)}^{2}}\right).$$

The eigenvalue associated with firm 1’s deviation from the boundary is

$$\lambda_{1}=1+\frac{v\alpha }{3}\left(c_{2}+c_{3}-c_{1}\right).$$

Thus, under symmetric costs, $\lambda_{1}=1+\frac{v\alpha c}{3}>1$, which explains the instability of $E_{1}$. By contrast, if the inactive firm has a sufficiently high marginal cost, namely

$$c_{2}+c_{3}<c_{1}<c_{2}+c_{3}+\frac{6}{v\alpha },$$

then the deviation of firm 1 from the boundary can be locally stable. The remaining two-firm active subsystem is locally stable when

$$\frac{w{\left(c_{3}-c_{2}\right)}^{2}}{4c_{2}c_{3}}<1.$$

Therefore, boundary equilibria may become economically meaningful and locally stable in asymmetric-cost regimes, especially when one firm is much less efficient than its active rivals. A full asymmetric-cost quantum triopoly model would change the equilibrium conditions and stability thresholds, and is left for future research. This possibility is consistent with studies emphasizing the role of cost asymmetry and isoelastic demand in Cournot-type dynamics [25,39,40].

To study the local stability of the interior equilibrium, we substitute $E_{2}$ into the Jacobian and obtain $J(E_{2})$ in Equation (22).

(22) $$J\left(E_{2}\right)=\left(\begin{array}{ccc} 1-\frac{2vc\alpha }{9}\; & -\frac{vc\alpha }{18}\; & -\frac{vc\alpha }{18}\\ -\frac{1}{4}\; & 0\; & -\frac{1}{4}\\ -\frac{1}{4}\;w\; & -\frac{1}{4}\;w\; & 1-w \end{array}\right).$$

Let the characteristic polynomial of $J(E_{2})$ be written as

(23) $$\lambda^{3}+A_{1}\lambda^{2}+A_{2}\lambda +A_{3}=0,$$

where

$$\begin{array}{cc} A_{1}= & \frac{2vc\alpha }{9}-2+w,\\ A_{2}= & \frac{5vcw\alpha }{24}-\frac{17vc\alpha }{72}-\frac{17w}{16}+1,\\ A_{3}= & \frac{w}{16}-\frac{vcw\alpha }{48}+\frac{vc\alpha }{72}. \end{array}$$

According to the Jury criterion, the quantum Nash equilibrium point $E_{2}$ is locally asymptotically stable if [12,13,26]

(24) $$\left\{\begin{array}{cc} (i)\; & 1+A_{1}+A_{2}+A_{3}>0,\\ (ii)\; & 1-A_{1}+A_{2}-A_{3}>0,\\ (iii)\; & 1-A_{2}+A_{1}A_{3}-{A_{3}}^{2}>0,\\ (iv)\; & 3-A_{2}>0. \end{array}\right.$$

**Proposition** **2.** *The quantum Nash equilibrium point $E_{2}$ is locally asymptotically stable provided that*

(25) $$v<\;\frac{576-306w}{(68-33w)c\alpha }.$$

**Proof.**  For v>0, c>0, γ≥0 and w∈(0,1), conditions (i) and (iv) of the Jury criterion are automatically satisfied. Indeed, one has

$$\begin{array}{cc} \left(i\right)\; & 1+A_{1}+A_{2}+A_{3}=\frac{3vcw\alpha }{16}>0,\\ \left(\mathrm{iv}\right)\; & 3-A_{2}=\frac{2vc\alpha (1-w)}{9}+\frac{vcw\alpha }{72}+\frac{w}{16}+\frac{vc\alpha }{72}+w+2>0. \end{array}$$

Next, consider condition (ii). After algebraic simplification, it can be written as

(26) $$\begin{array}{cc} \; & 1-A_{1}+A_{2}-A_{3}=4-\frac{17w}{8}-\left(\frac{17}{36}-\frac{11w}{48}\right)vc\alpha >0. \end{array}$$

Hence, condition (ii) holds whenever

(27) $$v<\;\frac{576-306w}{(68-33w)c\alpha }.$$

Finally, for condition (iii), direct simplification gives

(28) $$1-A_{2}+A_{1}A_{3}-{A_{3}}^{2}=\beta_{1}v^{2}+\beta_{2}v+\beta_{3},$$

where

$$\begin{array}{c} \beta_{1}=\frac{60-84w-9w^{2}}{20736}c^{2}\alpha^{2},\;\;\beta_{2}=\frac{720-486w-63w^{2}}{3456}c\alpha ,\;\;\beta_{3}=\frac{15}{16}w+\frac{15}{256}w^{2}. \end{array}$$

For w∈(0,1), the linear coefficient and the constant term are both positive. If $60-84w-9w^{2}\geq 0$, condition (iii) is immediately satisfied for v>0. If $60-84w-9w^{2}<0$, the above expression is a concave quadratic function of *v*. Since its value at v=0 is $\beta_{3}>0$, it remains to check its value at the upper boundary $v_{c}=\frac{576-306w}{(68-33w)c\alpha }$. Evaluating it at $v=v_{c}$, we obtain

$${\left.\left(1-A_{2}+A_{1}A_{3}-A_{3}^{2}\right)\right|}_{v=v_{c}}=-\frac{3\left(23w^{2}+112w-320\right)\left(597w^{2}-2304w+2432\right)}{256{\left(33w-68\right)}^{2}}>0,$$

where the inequality follows directly from w∈(0,1). Hence, condition (iii) holds for all $0<v<v_{c}$. Combining conditions (i)–(iv), the Jury criterion is satisfied whenever $v<\frac{576-306w}{(68-33w)c\alpha }$. Therefore, $E_{2}$ is locally asymptotically stable under the condition in Equation (25). □

## 4. Numerical Simulations

To further investigate the complex dynamical behavior of the heterogeneous quantum Cournot triopoly, we carry out a series of numerical simulations in this section. In particular, we examine the stability region of the interior equilibrium, the bifurcation evolution with respect to the adjustment speed, the emergence of chaotic attractors, and the sensitive dependence on initial conditions. The numerical simulations are performed for initial conditions in the feasible interior region, and the reported dynamics are interpreted as the behavior of the positive interior branch of the heterogeneous quantum Cournot triopoly.

### 4.1. Dynamics of the Stability Region

To clarify how the local stability of the Nash equilibrium $E_{2}$ depends on the key parameters, we linearize the discrete-time map at $E_{2}$ and apply the Jury stability criterion to obtain the admissible parameter constraints for local stability. These inequalities define the stability region of $E_{2}$, i.e., the set of parameter values for which $E_{2}$ is locally asymptotically stable.

**Figure 1:**
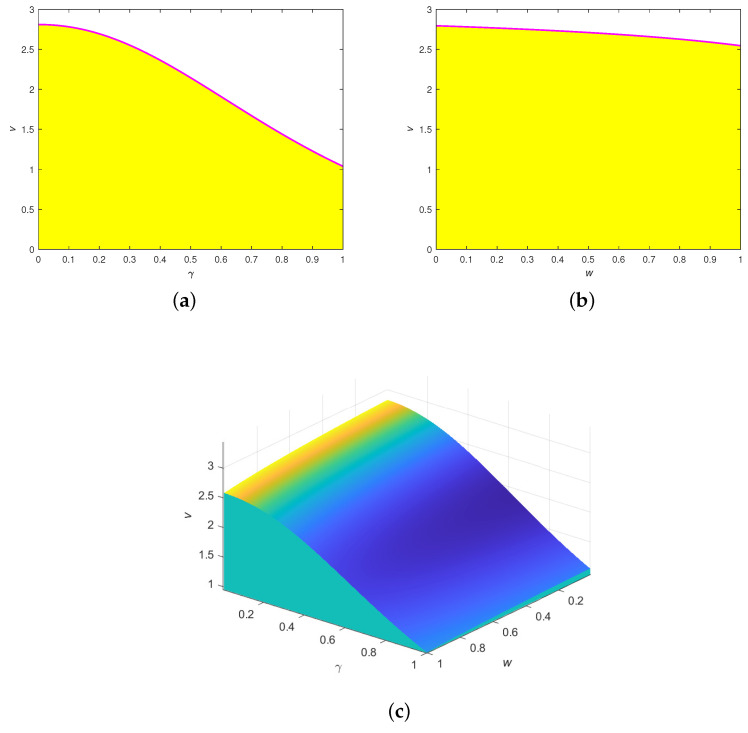
Stability region of the equilibrium $E_{2}$ obtained from the Jury conditions. (**a**) Stability domain in the (γ,v) plane for w=0.1 and c=1. (**b**) Stability domain in the (w,v) plane for γ=0.1 and c=1. (**c**) Critical surface $v_{c}\left(\gamma ,w\right)$ for c=1.

Figure 1 depicts the geometry of the stability domain in several representative parameter planes. In all panels, the yellow region corresponds to local asymptotic stability of $E_{2}$, while the magenta curve indicates the stability boundary. For clarity, we fix c=1 and visualize the stability structures in the (γ,v) and (w,v) planes, together with the corresponding critical threshold of the adjustment speed. Specifically, $v_{c}\left(\gamma ,w\right)$ denotes the critical adjustment speed, namely the largest value of *v* for which $E_{2}$ remains locally asymptotically stable for given (γ,w); thus, stability requires $0<v<v_{c}\left(\gamma ,w\right)$.

As shown in Figure 1a, for w=0.1 the stability region in the (γ,v) plane shrinks markedly as γ increases. In particular, the stability boundary $v=v_{c}\left(\gamma ,w\right)$ decreases monotonically with γ, leading to a substantial reduction of the stable domain. This implies that stronger entanglement narrows the admissible range of the adjustment speed, thereby lowering the stability threshold and making the system more susceptible to bifurcations and subsequent complex dynamics.

Figure 1b reports the stability region in the (w,v) plane for γ=0.1. Compared with its strong dependence on γ, the stability boundary varies only moderately with *w*, and the stable area remains relatively large across a wide range of *w*. This indicates that, under the present parameterization, the impact of the adaptive parameter *w* on local stability is weaker than that of the entanglement level γ: the adjustment speed *v* plays the dominant role in determining whether $E_{2}$ is stable, whereas variations in *w* mainly cause mild shifts of the critical threshold.

The quantitative effect of *w* can also be seen directly from the critical threshold $v_{c}=\;\frac{576-306w}{(68-33w)c\alpha }.$ For 0<w<1, its derivative with respect to *w* is $\frac{\partial v_{c}}{\partial w}=-\frac{1800}{{\left(68-33w\right)}^{2}c\alpha }<0,$ which confirms that a larger adaptive coefficient lowers the admissible adjustment speed. Under the baseline values c=1 and γ=0.1, $v_{c}\left(w\right)$ decreases from $v_{c}\left(0\right)\approx 2.795$ to $v_{c}\left(1\right)\approx 2.545$. Representative values are $v_{c}\left(0.25\right)\approx 2.758$, $v_{c}\left(0.5\right)\approx 2.710$, and $v_{c}\left(0.75\right)\approx 2.643$. Therefore, a larger *w* has a destabilizing effect, but this effect is relatively moderate.

To provide an overall view of the stability threshold, Figure 1c presents the critical surface $v=v_{c}\left(\gamma ,w\right)$. The three-dimensional plot shows that the critical value $v_{c}$ decreases as either γ or *w* increases, with a noticeably steeper decline along the γ direction. This further confirms the pronounced role of entanglement in shaping the stability evolution. Consequently, maintaining local stability of $E_{2}$ requires choosing *v* sufficiently small, and in particular, larger γ necessitates a smaller adjustment speed in order to avoid loss of stability through bifurcation and the emergence of complex dynamics.

### 4.2. Bifurcation and Chaos Analysis

#### 4.2.1. Bifurcation Evolution with Respect to *v*

**Figure 2:**
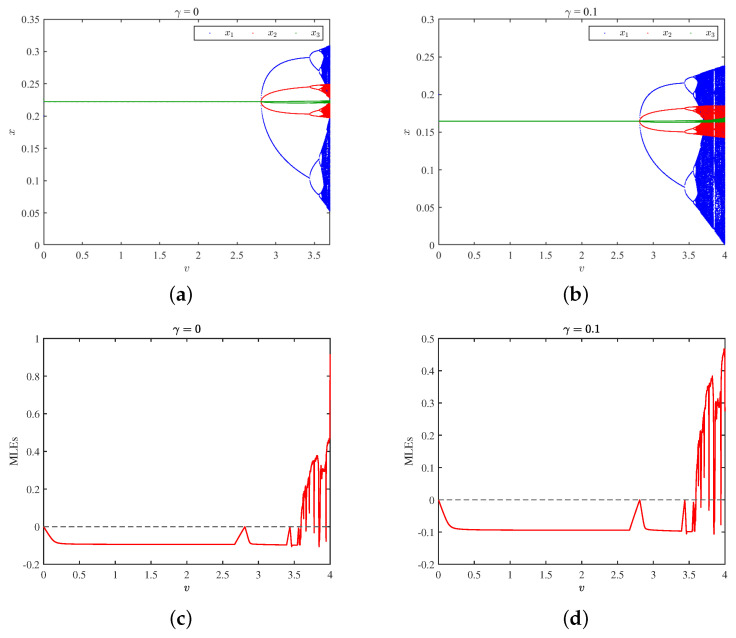
Bifurcation diagrams and maximum Lyapunov exponents (MLEs) versus the adjustment speed *v* with c=1 and w=0.1. (**a**) Bifurcation diagram for γ=0; (**b**) bifurcation diagram for γ=0.1; (**c**) maximum Lyapunov exponents for γ=0; (**d**) maximum Lyapunov exponents for γ=0.1. The dashed horizontal line in panels (**c**,**d**) indicates MLE=0.

To further explore the dynamical evolution of the system with respect to the adjustment speed *v*, we plot bifurcation diagrams of the state variables $x_{1}$, $x_{2}$, and $x_{3}$ versus *v*, and compute the maximum Lyapunov exponent (MLE) to diagnose chaotic behavior. The numerical results are summarized in Figure 2. Specifically, Figure 2a,b correspond to γ=0 and γ=0.1, respectively, while the remaining parameters are fixed at c=1 and w=0.1. For sufficiently small *v*, the trajectories converge to a unique steady state (the locally stable equilibrium $E_{2}$), indicating that slow adjustment leads the market to a stable outcome.

#### 4.2.2. Flip Bifurcation and Period-Doubling Cascade

As *v* increases, the equilibrium loses stability and the system undergoes its first bifurcation. As shown in Figure 2a, when γ=0 a flip (period-doubling) bifurcation occurs around the critical value v≈2.807, where the stable period-1 orbit destabilizes and a stable period-2 cycle emerges. With further increases in *v*, successive period-doubling bifurcations generate higher-order periodic orbits (e.g., period-4 and period-8), forming a typical period-doubling cascade. For γ=0.1, the first bifurcation occurs earlier at approximately v≈2.779, implying that a higher entanglement level reduces the stability margin and facilitates the transition from steady behavior to periodic oscillations. This observation is consistent with the stability analysis in Figure 1, where the critical threshold $v_{c}$ decreases monotonically as γ increases.

#### 4.2.3. Chaotic Bands and Periodic Windows

As *v* increases beyond subsequent thresholds, the iterates in the bifurcation diagrams expand from finitely many branches into dense bands, indicating the onset of chaos. In this regime, trajectories become aperiodic and highly sensitive to initial conditions, reflecting strong uncertainty in the adjustment process. Notably, periodic windows appear within the chaotic parameter range: over certain intervals of *v*, the chaotic bands are interrupted and the system temporarily returns to low-period oscillations. This alternation between chaotic and periodic dynamics is a well-known feature of nonlinear discrete-time maps and implies that small parameter perturbations may switch the system between predictable and unpredictable behaviors.

#### 4.2.4. Verification by Maximum Lyapunov Exponents

To corroborate the dynamical features observed in the bifurcation diagrams, the MLEs are computed and plotted in Figure 2c,d. For small *v*, the MLE is negative, indicating convergence to a stable equilibrium or a stable periodic orbit. As *v* increases, the MLE approaches zero at bifurcation points and becomes positive over certain parameter intervals, which confirms the emergence of chaotic dynamics. In particular, for γ=0.1 the MLE turns positive at a smaller value of *v* and remains positive over wider intervals, suggesting that stronger entanglement not only accelerates the loss of stability but also enlarges the parameter range associated with complex fluctuations and chaos. Therefore, *v* serves as a key bifurcation parameter governing the transition from stability to chaos, while increasing γ amplifies this destabilizing tendency.

### 4.3. Strange Attractor

**Figure 3:**
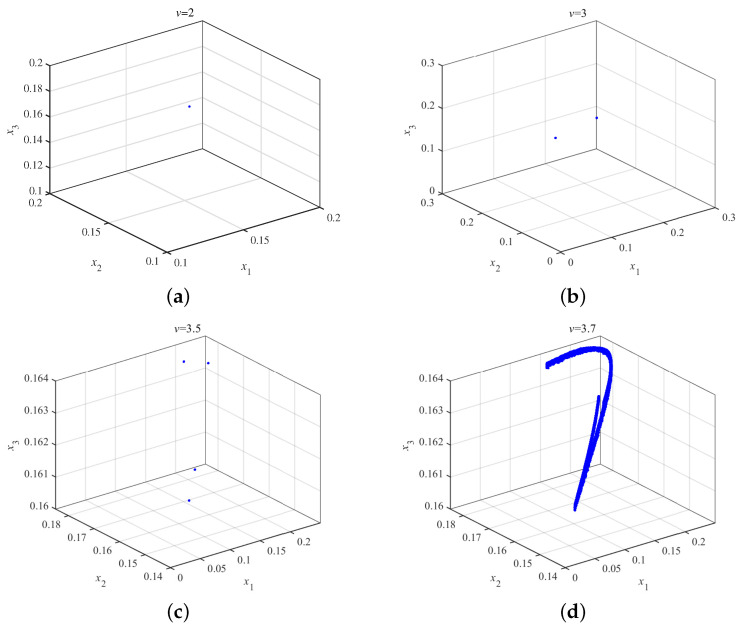
Phase portraits of the asymptotic dynamics in the three-dimensional state space for γ=0.1, c=1, and w=0.1. (**a**) v=2: stable fixed point. (**b**) v=3: period-2 orbit. (**c**) v=3.5: period-4 orbit. (**d**) v=3.7: strange attractor (chaos).

To illustrate the asymptotic dynamics more clearly, we plot the phase portraits in the three-dimensional state space $\left(x_{1},x_{2},x_{3}\right)$ for representative values of the adjustment speed *v*, as shown in Figure 3. In the simulations, a sufficient number of transient iterations are discarded, and only the remaining iterates are retained to depict the long-run dynamical behavior.

As shown in Figure 3a, when v=2 the trajectory converges to a single fixed point, indicating that the equilibrium $E_{2}$ is locally stable and the adjustment process drives the system toward a steady state. When *v* increases to v=3 (Figure 3b), the fixed point loses stability and the orbit approaches a period-2 cycle, represented by two distinct points in phase space, which is consistent with the flip bifurcation identified in the bifurcation diagrams. With a further increase to v=3.5 (Figure 3c), the long-run orbit consists of four points, revealing a stable period-4 cycle and confirming the period-doubling route to complexity.

For v=3.7 (Figure 3d), the trajectory no longer settles on a finite set of points. Instead, it fills a bounded region with a highly intricate geometric structure, which is consistent with the presence of a strange attractor. In this regime, the system exhibits chaotic motion characterized by aperiodic oscillations and strong sensitivity to initial conditions, which is commonly associated with the stretching-and-folding mechanism in nonlinear discrete-time dynamics. Overall, increasing *v* strengthens the effective feedback in the adjustment process, driving the system from a stable equilibrium to periodic oscillations and eventually to chaotic dynamics associated with a strange attractor.

### 4.4. Sensitive Dependence on Initial Conditions

To further confirm the chaotic nature of the system in the chaotic parameter regime, we investigate the sensitive dependence on initial conditions, a fundamental characteristic of chaotic systems. In particular, two trajectories that start from nearly identical initial states are expected to diverge rapidly after a finite number of iterations, making long-term prediction practically impossible.

In the chaotic regime with γ=0.1, c=1, w=0.1, and v=3.7, we select the baseline initial condition $x^{\left(1\right)}\left(0\right)=\left(0.15,0.20,0.20\right)$. To examine sensitivity to different state variables, we construct three nearby initial conditions by perturbing one component at a time

$$x_{1}\left(0\right)\to x_{1}\left(0\right)+10^{-4},\;x_{2}\left(0\right)\to x_{2}\left(0\right)+10^{-4},\;x_{3}\left(0\right)\to x_{3}\left(0\right)+10^{-4}.$$

**Figure 4:**
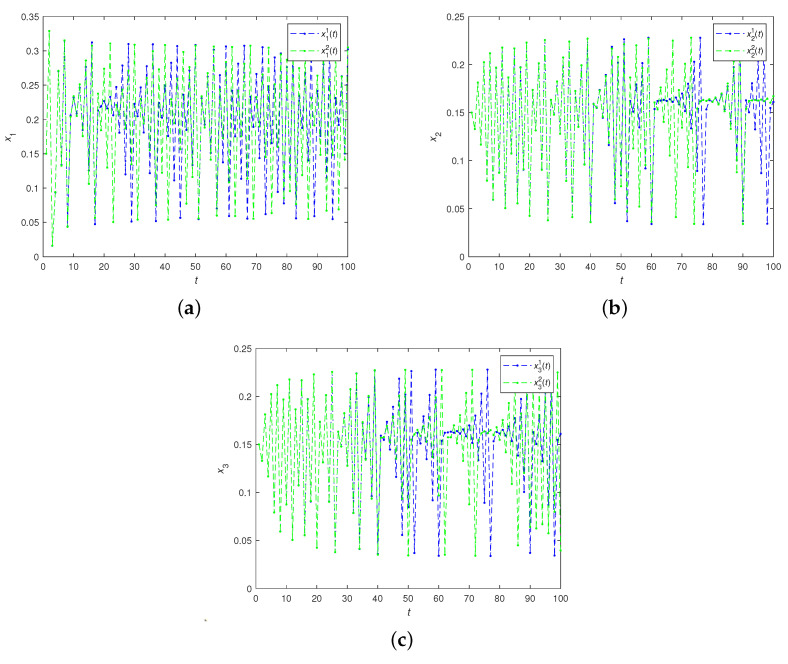
Sensitive dependence on initial conditions in the chaotic regime γ=0.1, c=1, w=0.1, and v=3.7. In each panel, two trajectories are generated from $x^{\left(1\right)}\left(0\right)=\left(0.15,0.20,0.20\right)$ and a nearby initial condition $x^{\left(2\right)}\left(0\right)$ obtained by perturbing one component: (**a**) $x_{1}\left(0\right)\to x_{1}\left(0\right)+10^{-4}$; (**b**) $x_{2}\left(0\right)\to x_{2}\left(0\right)+10^{-4}$; (**c**) $x_{3}\left(0\right)\to x_{3}\left(0\right)+10^{-4}$.

Figure 4 compares the time evolutions of the two trajectories. Initially, the orbits remain nearly indistinguishable, but as the iterations proceed, the discrepancy between the two trajectories grows rapidly, leading to a clear separation. This behavior demonstrates the sensitive dependence on initial conditions, which is a hallmark of chaotic dynamics. The positive maximum Lyapunov exponent calculated for this parameter setting further confirms the presence of chaotic motion.

Furthermore, the three panels in Figure 4 show that the divergence occurs regardless of whether the perturbation is applied to $x_{1}$, $x_{2}$, or $x_{3}$. This indicates that the sensitivity is not limited to a specific variable but rather results from the overall nonlinear coupling of the system. Therefore, as the adjustment speed *v* increases, the market dynamics become highly sensitive to initial conditions, leading to significant uncertainty and unpredictability in the system’s behavior.

## 5. Chaos Control

When the system enters a chaotic regime, its trajectories become aperiodic and highly sensitive to small perturbations, which reduces predictability and may hinder managerial planning and regulatory intervention. To suppress such fluctuations, we introduce a control parameter k≥0 into the boundedly rational adjustment rule of firm 1. Similar feedback-based control ideas have been widely used in Cournot-type nonlinear systems [43,44,45]. In the present model, the control parameter rescales only the adjustment increment of firm 1, leading to the controlled system

(29) $$\left\{\begin{array}{c} x_{1}\left(t+1\right)=x_{1}\left(t\right)+\frac{vx_{1}\left(t\right)}{1+k}\left(\frac{e^{-3\gamma }\left(x_{2}\left(t\right)+x_{3}\left(t\right)\right)}{{\left(x_{1}\left(t\right)+x_{2}\left(t\right)+x_{3}\left(t\right)\right)}^{2}}-\frac{c\alpha }{3}\right),\\ x_{2}\left(t+1\right)=\sqrt{\frac{3e^{-3\gamma }\left(x_{1}\left(t\right)+x_{3}\left(t\right)\right)}{c\alpha }}-x_{1}\left(t\right)-x_{3}\left(t\right),\\ x_{3}\left(t+1\right)=\left(1-w\right)x_{3}\left(t\right)+w\left(\sqrt{\frac{3e^{-3\gamma }\left(x_{1}\left(t\right)+x_{2}\left(t\right)\right)}{c\alpha }}-x_{1}\left(t\right)-x_{2}\left(t\right)\right). \end{array}\right.$$

When k=0, system (29) reduces to the original uncontrolled system. When k>0, the effective adjustment speed of firm 1 becomes v/(1+k), so a larger *k* weakens the nonlinear feedback generated by gradient-based updating.

The control parameter does not change the equilibrium set. Thus, the role of *k* is to modify the local stability properties rather than the equilibrium structure. To examine the local effect of the control parameter, we linearize the controlled map at the quantum Nash equilibrium $E_{2}$. The corresponding Jacobian matrix is

(30) $$J_{k}\left(E_{2}\right)=\left(\begin{array}{ccc} 1-\frac{2vc\alpha }{9(1+k)} & -\frac{vc\alpha }{18(1+k)} & -\frac{vc\alpha }{18(1+k)}\\ -\frac{1}{4} & 0 & -\frac{1}{4}\\ -\frac{w}{4} & -\frac{w}{4} & 1-w \end{array}\right).$$

Compared with the uncontrolled Jacobian, all entries associated with the adjustment speed *v* are multiplied by 1/(1+k). Therefore, increasing *k* reduces the effective adjustment speed and can restore the Jury stability condition for $E_{2}$.

Since the control parameter replaces *v* by v/(1+k), the stability condition obtained in Proposition 2 can be directly applied to the controlled system. Hence, the controlled equilibrium $E_{2}$ is locally asymptotically stable whenever

$$\frac{v}{1+k}<v_{c}=\frac{576-306w}{(68-33w)c\alpha },$$

or equivalently,

$$k>k_{c}=\frac{(68-33w)c\alpha v}{576-306w}-1.$$

For the parameter values used in the numerical simulation, namely c=1, γ=0.1, w=0.1, and v=3.7, this gives $k_{c}\approx 0.3304$.

The control parameter is applied to the boundedly rational firm because the adjustment speed *v* is the main bifurcation parameter and directly determines the strength of the gradient-feedback term. Thus, rescaling *v* provides a minimal intervention that preserves the equilibrium set and leaves the naïve and adaptive firms’ updating rules unchanged. In principle, one may introduce an additional control parameter $k_{w}$ to rescale the adaptive parameter as $w/(1+k_{w})$. More generally, if *v* and *w* are controlled by two independent parameters $k_{v}$ and $k_{w}$, the stability condition can be written as

$$\frac{v}{1+k_{v}}<\frac{576-306w/(1+k_{w})}{\left(68-33w/(1+k_{w})\right)c\alpha }.$$

If only *w* is rescaled while *v* remains unchanged, stabilization cannot be achieved for the chaotic case v=3.7 considered here. Indeed, as $w/(1+k_{w})\to 0$, the maximum stability threshold is $v_{c}\left(0\right)=\frac{576}{68c\alpha }\approx 2.795,$ which is still smaller than 3.7. Therefore, controlling *v* is more direct and effective in the present setting.

**Figure 5:**
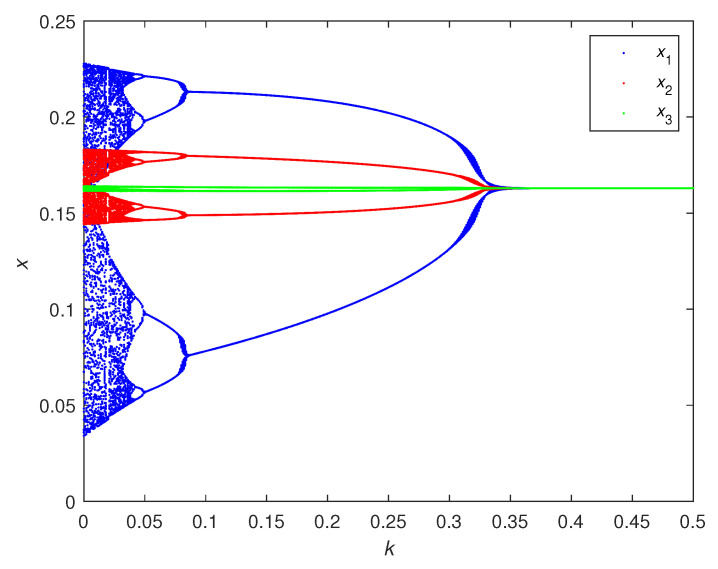
Bifurcation diagram of $x_{1}$, $x_{2}$, and $x_{3}$ versus the control parameter *k*. The diagram is computed from Equation (29) after discarding transient iterations. The parameters are fixed at c=1, γ=0.1, w=0.1, and v=3.7.

Numerical simulations further confirm the effectiveness of the proposed control mechanism. Figure 5 shows the bifurcation diagrams of $x_{1}$, $x_{2}$, and $x_{3}$ with respect to the control parameter *k*. For small *k*, the trajectories remain chaotic. As *k* increases, the chaotic bands gradually shrink and the system passes through periodic oscillations before converging to a stable fixed point. In particular, for the chosen parameter set, convergence is restored once *k* exceeds approximately 0.3304, which is consistent with the analytical threshold derived above. These results show that the proposed control parameter can suppress chaotic fluctuations without altering the equilibrium structure of the quantum Cournot triopoly.

## 6. Conclusions

This paper investigated the complex dynamics and stabilization of a continuous-variable quantum Cournot triopoly with heterogeneous expectations and isoelastic demand. By incorporating the Li–Du–Massar quantization scheme into a three-firm Cournot framework, we constructed a discrete-time nonlinear system in which the firms adopt boundedly rational adjustment, naïve expectations, and adaptive expectations, respectively. The quantum boundary equilibrium and the unique interior quantum Nash equilibrium were derived explicitly, and the local stability conditions of the interior equilibrium were obtained by linearizing the three-dimensional map and applying the Jury criterion.

The analytical and numerical results show that quantum entanglement plays a dual role in the proposed model. On the one hand, stronger entanglement increases the symmetric equilibrium payoffs in the static quantum Cournot game. On the other hand, it reduces the admissible range of the adjustment speed and shrinks the local stability domain of the interior equilibrium. Therefore, entanglement may enhance equilibrium performance while simultaneously increasing dynamical fragility. This finding highlights an important trade-off in quantum oligopoly dynamics: the same quantum correlation that improves equilibrium outcomes may also make the adjustment process more vulnerable to instability, bifurcation, and chaos.

The numerical simulations further demonstrate that the adjustment speed of the boundedly rational firm is a key source of complex dynamics. When the adjustment speed is relatively small, the system converges to the interior quantum Nash equilibrium. As the adjustment speed increases, the equilibrium loses stability through a flip bifurcation, followed by period-doubling cascades and chaotic motion. The emergence of chaos was identified through bifurcation diagrams and phase portraits, and further confirmed by positive maximum Lyapunov exponents and sensitive dependence on initial conditions. These results indicate that excessively fast local adjustment can amplify nonlinear feedback and generate unpredictable market fluctuations.

To suppress chaotic fluctuations, we introduced a control parameter for the boundedly rational firm. The proposed control mechanism rescales the effective adjustment speed without changing the equilibrium set of the system. The analysis of the controlled system shows that a sufficiently large control parameter can restore the local stability condition of the interior equilibrium, and the numerical bifurcation analysis confirms convergence to a stable fixed point once the control intensity exceeds the critical threshold. From a managerial perspective, the control parameter can be interpreted as a mechanism of moderate adjustment or regulatory smoothing, suggesting that reducing overly aggressive strategic adjustment may help stabilize market dynamics without altering the equilibrium structure.

Overall, this study shows how quantum entanglement, heterogeneous information-processing rules, and nonlinear demand jointly reshape the stability, complexity, and controllability of a nonlinear economic game. The results provide a complexity-oriented perspective on quantum oligopoly dynamics and enrich the literature on quantum games, heterogeneous Cournot competition, and chaos control. Future research may extend the present framework by considering quantum noise, asymmetric costs, capacity constraints, alternative expectation rules, and more general isoelastic-demand functions. It would also be valuable to compare different control schemes, such as delayed feedback control, state feedback control, and adaptive control, in order to evaluate their relative effectiveness in stabilizing quantum economic dynamics.

## Data Availability

No external datasets were used in this study. The numerical simulation data generated and analyzed during the current study are available from the corresponding author upon reasonable request.
